# Innate Immune Response in Orthopedic Implant Failure

**DOI:** 10.26502/josm.511500073

**Published:** 2022-12-05

**Authors:** Rajiv Supra, Devendra K Agrawal

**Affiliations:** 1 College of Osteopathic Medicine, Touro University, Henderson, Nevada; 2 Department of Translational Research, Western University of Health Sciences, 309 E. Second Street, Pomona, California 91766-1854, USA

**Keywords:** Inflammation, Innate immune response, Orthopedic implants, Prosthetic debris

## Abstract

The total joint replacement is recognized as one of the most effective medical arbitrations leading to increased mobility, pain relief, and an overall restored function of the joint. Unfortunately, prosthetic debris accumulates after long-term wear of the implant leading to activation of the innate immune response and periprosthetic osteolysis. Understanding the intricate biological mechanisms underlying the innate immune response to implant debris would support the development of novel pharmacological treatments to prolong the life span of the implant. This article provides a detailed description on the role of the innate immune system in response to implant debris, emphasizing the most recent research and outstanding questions. Furthermore, a critical discussion is presented on the novel pharmacological treatments currently under investigation to prevent implant failure.

## 1. Introduction

As successful orthopedic surgical implants increase over the years, the vast immunological complications associated with them become imperative to understand. Examples of such surgical interventions include the total knee arthroplasty, which is projected to increase by 601% from 2005 to 2030 [1]. Although success rates reaching greater than 90% for total knee arthroplasty have been documented [2], over one million replacements each year are projected to fail after 15 to 25 years of use due to subtle inflammation [3]. Periprosthetic inflammation is due to macrophage activation and response to implant byproducts which result in the activation of osteoclasts and osteolysis, leading to aseptic loosening [4]. Aseptic loosening (no infection) has been responsible for more than 70% of hip revisions and over 44% of knee revisions [5,6]. Although biomaterials coat implants such as ceramics, polymers, and metals, repeated movements of bearing surfaces produce wear debris [4]. Wear particles such as metal ions trigger an exacerbated immune response [7]. Analysis of tissues around implants retrieved during repeat surgeries of failed total joint replacements revealed Ultra-High Molecular Weight Polyethylene (UHMWPE) debris as the most frequently observed type of debris around hip, knee, and shoulder total joint replacements [8]. Particles from UHMWPE wear debris were retrieved and quantified from 123 tissue samples from failed orthopedic implants and showed a mean size of 0.5 μm in diameter with 90% of the debris particles being reportedly less than 1 μm in diameter [9,10]. Particles less than 20 μm are phagocytosed by infiltrating macrophages, and overall, the immune response to polyethylene is predominated by the response of innate immune system to polymer wear debris [11].

Substantial evidence suggests the involvement of cells, such as osteoblasts, lymphocytes, fibroblasts, and others, in the underlying mechanisms of osteolysis. The inflammatory response is mainly driven by the innate immune system, specifically macrophages [12,13]. The role of the innate immune system in maintaining homeostasis includes clearing cellular debris and defending against foreign invaders. Macrophages do not require exposure to antigens to initiate a response against foreign material. These cells protect the host against potentially harmful stimuli and are the first line of protection against pathogenic microbes [14]. Upon activation, macrophages release a plethora of cytokines and inflammatory mediators in the joint that lead to the maturation, differentiation, and recruitment of osteoclasts [15]. Initial activation of the innate immune system in response to implants and the chronic inflammation that results from it ultimately leads to long-term loosening and failure of the implant.

Our knowledge of orthopedic implantology and the potential infectious consequences have been extensively investigated, but non-infectious immune rejection reactions against implanted materials still need to be thoroughly examined. The main focus of this review is to understand the role of the innate immune system in response to orthopedic implant failure in relation to debris accumulation.

## 2. Innate Immune response to wear debris particles

**Figure 1:**
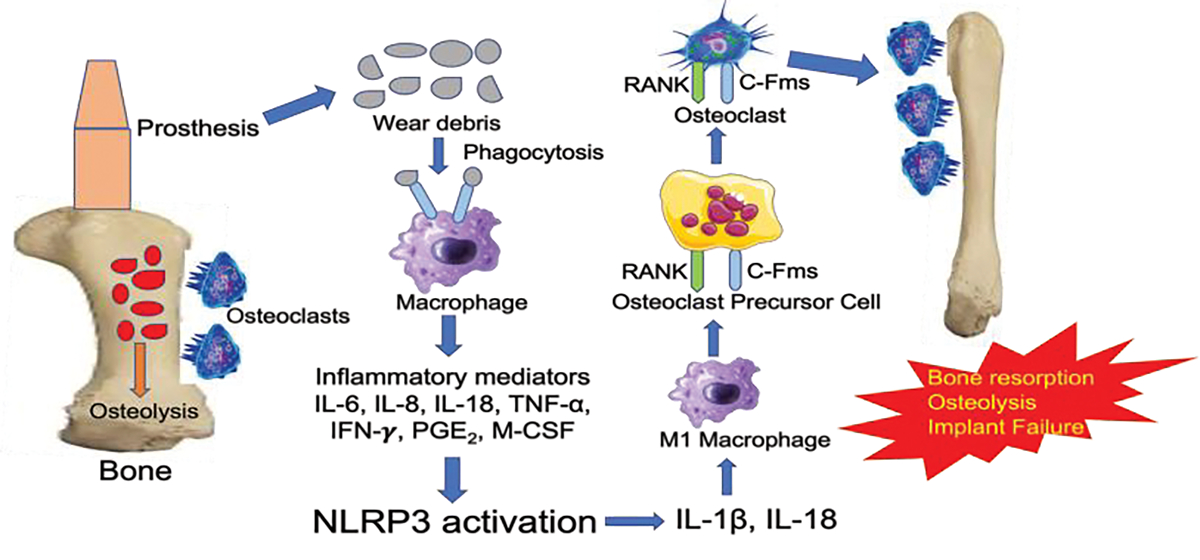
Implant debris-induced macrophage activation and the innate immune responses leading to bone resorption, osteolysis, and implant failure. The wear debris from the implant are recognized as foreign particles that are phagocytosed by macrophages. These macrophages become activated to release inflammatory mediators that activate downstream NLRP3 inflammasome to release IL-1β and IL-18. These cytokines activate inflammatory M1 macrophages to induce the differentiation of osteoclast precursor cell into osteoclasts. The osteoclast then binds to bone resulting in bone resorption, osteolysis, and implant failure. NLRP3, NOD-, LRR- and pyrin domain-containing protein 3; RANK, regulated by receptor activator of nuclear factor-κB; c-Fms, receptor for colony stimulating factor-1.

Implant debris-induced inflammation has been a central cause of long-term implant failure. Aseptic implant failure due to inflammation is responsible for over 70% of hip arthroplasty revisions [16]. Peri-implant osteolysis (local bone loss) results from inflammatory responses from small wear particles less than 10 μm in diameter [17]. Macrophages are among the most plastic cells in the body with different functional states and actions. Two polarized phenotypes have been studied and referred to as M1 or activated and inflammatory macrophages and M2 or alternatively activated macrophages [18–20]. In response to wear particles, the M1 macrophages release inflammatory cytokines such as IL-8, tumor necrosis factor-alpha (TNF-α), macrophage colony-stimulating factor (M-CSF), IL-6, and pro-osteoclastic factors, including receptor activator of nuclear factor kappa B ligand (RANKL) (17) (Figure 1). Indeed, the response to implant debris leads to osteoclast activation through up-regulation of RANKL, IL-6, and TNF-α released from macrophages. Cytokine release in response to wear particles suppresses osteoprotegerin (OPG) from being expressed by osteoblasts [15]. RANKL binds to RANK expressed on osteoclast precursors activating pathways such as Mitogen-Activated Protein Kinase (MAPK), leading to osteoclastogenesis and bone resorption, ultimately leading to implant loosening and failure [21].

Macrophage response to wear particles and subsequent release of cytokines is determined by Pattern Recognition Receptors (PRRs) [2,12]. PRR recognizes a substantial number of stimuli, including Pathogen-Associated Molecular Patterns (PAMPs) and Damage-Associated Molecular Patterns (DAMPs). While it is known that PAMPs are materials derived from various infectious organisms, DAMPs are produced during times of homeostasis disturbance and tissue damage [22–24]. PRRs are divided into Toll-like Receptors (TLRs) and transmembrane proteins such as C-type lectin receptors. The retinoic acid-inducible protein (RIG) -1- like receptors as well as the NOD-Like Receptors (NLRs) are located intracellularly [25]. Evidence suggests that DAMPs and TLRs are central in macrophage reactivity to implant particles and processes including bone catabolism, hypoxia, and apoptosis [26].

Pathways activated by TLRs are classified into myeloid differentiation factor 88 (MyD88)-dependent and My-D88-independent. Signaling by MyD88 results in the activation of nuclear factor kappa-light-chain-enhancer of activated B cells (NF-kB) and production of TNF-α, IL-1, and IL-12 [27]. TLRs are widely expressed by macrophages that infiltrate tissues surrounding the implants of patients with aseptic loosening [28]. Inhibition of MyD88 signaling in in vitro cultures of macrophages decreased the inflammatory reaction to Polymethylmethacrylate (PMMA) particles while MyD88 knockout mice showed an overall reduction in the inflammatory response to PMMA particles suggesting potential therapeutic targets in this pathway [29].

The NOD, LRR, and pyrin domain-containing protein 3 (NLRP3/NALP3) inflammasome is an intracytoplasmic sensor that assembles into a protein complex within inflammatory cells upon infection (PAMPs) or tissue damage (DAMPs), leading to maturation of inflammatory cytokines such as IL-18 and IL-1β [13,30,31]. Activation of the NLRP3 inflammasome pathway contributes to implant failure by responding to implant debris and requires a two-step process. The first step is the priming signal leading to NF-kB activation. The second is the oligomerization of NLRP3 by recruiting pro-caspase-1 via adaptor molecule apoptosis-associated speck-like protein containing a CARD (ASC) and cleavage of cytokine precursors leading to release of mature forms of IL-18 and IL-1β [32]. Recent research revealed polarization toward the M1 phenotype for macrophages responding to implant debris [33]. Therefore, the biologically active wear particles generated from orthopedic implants influence the innate immune response through their size, amount, and rate of production, all of which are integral factors in the inevitable demise of the implant in the long term [12,34,35]. Once macrophages have phagocytosed implant debris, the NLRP3 inflammasome pathway becomes activated, and the cytokines released include IL-1β, IL-10, IL-11, IL-15, TNF-α, transforming growth factor-α, GM-CSF, platelet-derived growth factor, and epidermal growth factor [13,16] (Figure 1).

Implant wear debris and its various chemical attributes induce different responses from macrophages. In the following sections, the uses of different materials in orthopedic joint implants are discussed along with the ways the innate immune system responds to them.

## 3. Complement System Activation Against Implant Materials

The complement system is a crucial component of the innate immune system and plays a crucial role in inflammation. The complement system recognizes DAMPs and is part of the humoral defense system of the innate immune system [23,36]. Activation of the complement primarily occurs in three different pathways which include the classical pathway, lectin pathway, and alternative pathway. The classical pathway is activated by antigen-antibody complexes and the C1 component of the complement system recognizes the immunoglobulin (Ig)G or IGM. This C1 component eventually cleaves C2 and C4. These split products generate a C3 converse (C4bC2a) that generates C3a and C3b from cleaving C3 [36]. The alternative pathway forms a C3 convertase (C3bBb) as well and enables a rapid reaction to DAMPs which further activates the complement system. The lectin pathway is initiated upon Mannose-Binding Lectins (MBL) recognizing carbohydrate residues abundant on bacterial cell membranes [36]. The C4 and C2 are cleaved, and a C3 convertase (C4bC2a) is formed, cleaving C3 into C3a and C3b. The C3a is a potent chemoattractant, while C3b binds foreign materials to induce their phagocytosis [36]. The C3b is a component of C3 and C5 convertases, the latter of which generates C5a, an anaphylatoxin. The C5b, the second split product, is a submission of the terminal complement complex (TCC, C5b-9). This complex is a membrane pore-forming structure that assembles on foreign surfaces to initiate complement-mediated destruction [37].

Orthopedic biomaterials are considered foreign substances and can activate the complement system [38]. Macrophages can recognize implant wear particles that have been complement-opsonized via their receptors. This leads to the transformation of macrophages into multinucleated Foreign Body Giant Cells (FBGCs), which are a significant component of immune reaction to orthopedic implant materials [39]. Oxygen-free radicals, inflammatory cytokines, and degrading enzymes are released from FBGCs, which all contribute to osteolysis [38,40]. The hallmark of orthopedic implant failure and peri-prosthetic osteolysis may be due to the abundance of FBGCs [40].

## 4. Immune Response Against UHMWPE

The most successful hip and knee replacements have used UHMWPE biomaterials which have been the gold standard due to their high wear resistance and biocompatibility relative to other materials [12,41]. Long chains of polyethylene makeup UHMWPE. A study evaluated the influence of different radiation conditions on the wear behavior of Vitamin E blended and six weeks artificially aged UHMWPE gliding components and revealed a higher wear resistance for the Gamma irradiated cross-linked UHMWPE [42]. Despite recent studies of decreased wear reduction rates using UHMWPE, the release of wear particles results in periprosthetic loosening in long-term implant use. Macrophages attempt to phagocytose the inert UHMWPE particles but fail to do so. The macrophages become activated and release inflammatory mediators that form a thick granulomatous tissue, abundant in activated macrophages [12,43]. Polyethylene particles can also activate the alternative pathway of the complement cascade due to the presence of C3a, C5b-9, and Bb in the synovial tissue retrieved from hip arthroplasty revisions carrying polyethylene implants [44].

## 5. Immune Response Against Metal Implant

The generation of metallic particles has become a matter of concern regarding the activation of the immune system and eventual aseptic loosening. Macrophages take up particles through pinocytosis and endocytosis and internalize larger particles through the lysosomal pathway [45]. Despite this immune response to metal particles, there have been fewer reported cases of osteolysis for Metal-on-Metal (MoM) implants compared to UHMWPE-coated implants [12,46]. This could be due to many metallic wear particles being smaller in size and volume than Metal on Polyethylene (MoP) bearings.

**Figure 2:**
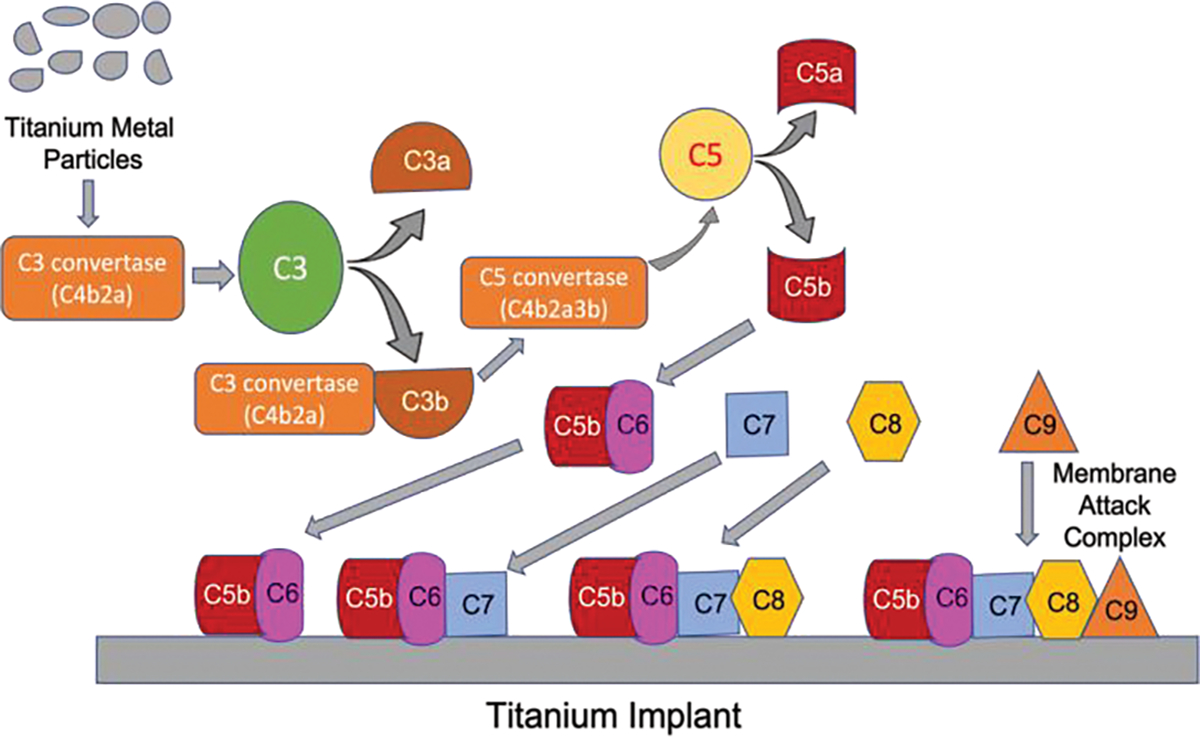
Activation of Complement system by titanium metal particles in Implant failure. The titanium metal debris activates C3 convertase (C4b2a) to cleave C3 into C3a and C3b. The resulting C3b binds to C3 convertase to become C5 convertase (C4b2a3b) which then cleaves C5 into C5a and C5b. C3a and C5a act as anaphylatoxins to induce an inflammatory response. C5b binds to other complement fragments starting with the binding to C6 to form C5b6 complex which then binds to C7 and C8 in a sequential manner. The C5bC7C8 complex binds to multiple molecules of C9 to form a membrane attack complex (C5b-9 complex). The complement fragments and membrane attack complex bind to the surface of the titanium implant resulting in the degradation of the implant and thus leading to implant failure.

Although biomaterials made of metals such as titanium have been deemed biocompatible, corrosion and subsequent particle release remain an issue [47]. Titanium metal particles can induce the activation of the complement system by activating C3b, C3a, and C5a by C3 and C5 cleavage of human plasma, as revealed through in vitro studies [48] (Figure 2). Surface modifications of Titanium implants were found to play a crucial role in the activation of the innate immune system. Studies revealed, for example, that C3 is preferentially bound to smoother titanium surfaces than rough surfaces [49]. Treating titanium surfaces with Ultraviolet (UV)-light, however, significantly reduced the activation of complement and subsequent inflammation [50]. Interestingly, UV-illumination of titanium enhanced early bone apposition to the implant in rat tibiae [51]. To further investigate the effects of complement on metal implants, in vivo implant anchorage and bone healing were studied upon implanting titanium screws in rate tibiae. The study revealed greater inflammation from complement activation and reduced bone formation without significant impact on bone anchorage [52]. Although these studies imply a rather negative effect of the innate immune system on titanium implants, recent studies have challenged these views. Osseointegration and inflammation were studied after rabbit femurs were implanted with titanium and subsequent foreign body reaction ensued expectantly. However, this reaction seemed to enhance osseointegration of the implant as new bone formation, and reduced bone resorption was detected. Additionally, the bone formation in the periimplant area seemed more mature than the sham bone introduced in rabbit femurs devoid of any biomaterials. Low C3 levels were observed and thought to contribute to decreased bone resorption [53].

The complex interplay between the immune system and different biomaterials for orthopedic implants has yet to be fully understood. Aseptic loosening secondary to wear particles is a serious health issue in orthopedics today and will continue to have an impact on patients in the coming decades. The initial response to joint implants is a sudden inflammatory response that progresses over many years, provoking many chemokines in the process. Many of these cytokines are involved in the differentiation of osteoclasts contributing to osteolysis. Clarifying the molecular details and chemokines involved in the inflammatory response against prosthetic wear debris will present an enormous therapeutic benefit when implementing potential therapies against these pathways.

## 6. Chemokines Involved in Biomaterial-Induced Inflammation

Chemokines are a central component of orthopedic implant failure and are expressed by fibroblasts, macrophages, and osteoblasts. Chemokines help enhance the migration of leukocytes to and from sites of implant debris accumulation. The chemokines specific to orthopedic implant aseptic loosening include monocyte chemotactic protein 1 (MCP-1), IL-8, MIP-1, CCL22/monocyte-derived chemokine (MDC), CCL17/thymus and activation-regulated chemokine (TARC) all of which have been implicated in implant debris reactivity [54] (Table 1).

### 6.1 Interleukin-8 (IL-8)

IL-8 is released from macrophages, mast cells, and endothelial cells. IL-8 is a well-established chemokine present in peri-implant tissues and is used as a biomarker for peri-implant osteolysis [55]. IL-8 recruits neutrophils and macrophages to the sites of inflammation caused by implant debris. However, the extent of IL-8 recruitment of neutrophils and the effects of implant long term is yet to be fully understood [56].

### 6.2 Monocyte Chemotactic Protein-1 (MCP-1)

Release of chemokines by macrophages in response to wear debris around the bone-implant interface may lead to chronic inflammation and implant failure. Peri-implant tissues from failed arthroplasties expressed chemokines such as MCP-1 (CCL2), MIP-1 α (CCL3), and MIP-1β (CCL4) [57]. Upon exposure to titanium and PMMA particles, fibroblasts increased the release of MCP-1 [58]. Studies also revealed MCP-1 to play a major role as a potential biomarker of osteolysis due to its presence in tissues surrounding failed total joint replacements [59]. Increased expression of MCP-1 was also noted in RAW 264.7 macrophage cells in response to UHMWPE and PMMA particles [55]. While controversy remains on whether antibodies blocking the MCP-1/CCR2 interaction is efficient at inhibiting macrophage recruitment in vitro [60], in vivo studies revealed a murine femoral implant model injected with MCP-1 recruited macrophages in the presence of UHMWPE particles. Interrupting the MCP-1/CCR2 axis seemed to decrease macrophage recruitment, potentially creating a viable strategy to mitigate macrophage migration to sites of implant debris [61]. However, there is a lack of sufficient in vivo studies to indicate whether interrupting the MCP-1/CCR2 interaction will prevent particle-induced activation of the immune system and subsequent implant failure.

## 7. Toll-Like Receptors and Aseptic Loosening

TLRs have been observed on many cells, including monocytes, macrophages, osteoclasts, and fibroblasts. Studies reveal that TLRs are found in peri-prosthetic tissues and play a significant role in implant failure [62]. Steel rods inserted into mouse femurs revealed titanium particles inducing the expression of TLR-1 and TLR-2. It was found that only TLR-1 expression increased in the presence of titanium particles [63]. TLR-2 was observed on macrophages in aseptic periprosthetic tissues in total hip implants [64]. Other studies also revealed an increase in the expression of TLR-2 with titanium particles coated with LPS. TLR-4, TLR-5, and TLR-9, however, were all decreased, suggesting some sort of self-protective mechanism from inflammation [64]. Greenfield et al. [65] used a murine calvarial model of particle-induced osteolysis and found decreased osteolysis in TLR2−/− mice compared to wild-type mice. TLR2−/− macrophages had reduced levels of TNF-α in the presence of titanium particles. The in vivo and in vitro data presented strongly reinforce the role of TLRs in orthopedic implant failure.

TLR-4, a receptor for Lipopolysaccharide (LPS), has become critical in understanding aseptic loosening. In tissues around loosened replacement implants, there was an increase in TLR-4 expression [66]. When TLR-4 was mutated, the inflammatory response and subsequent osteolysis decreased when exposed to wear particles [67]. TLR-4 knockout mice displayed decreased osteolysis and revealed the major role of TLR-4 in aseptic loosening. Hao et al. [68] revealed that UHMWPE particles induced TLR-4 and HSP60 expression on monocytes. IL-1β, IL-6, and TNF-α are released when HSP60 binds TLR-4. Titanium particles similarly can induce inflammatory cytokines and osteolysis from TLR-4 reacting with wear particles with adherent LPS [67]. LPS has been shown to be present in tissues around loosened implants [68]. However, it remains unclear whether LPS in periprosthetic tissues is required for the initiation of the inflammatory response against wear particles. Hirayama et al. [64] found that LPS decreased the expression of TLR-4 compared to debris particles without LPS. This alludes to a self-protective mechanism where macrophages can easily recognize LPS-coated debris particles via TLR-4. After the initiation of the inflammatory response against debris particles, TLR-4 was eventually downregulated to protect the host from excessive harmful response. Reduced mRNA expression of TLR-4 was seen in RAW 264.7 cells stimulated with titanium debris particles in vitro [63]. TLR expression was downregulated through paracrine inflammatory cytokines to prevent excessive host response [63]. It was noted that when TLR-4 knockout macrophages were challenged with wear debris, similar levels of TNF-α were recorded compared to wild-type macrophages. Macrophages that lacked TLR-4 and TLR-2, the osteolysis in vivo was only slightly inhibited. It seemed that early inflammatory reactions against periprosthetic debris were TLR-dependent while long-term, osteolysis was only partially TLR-dependent [65].

Further research has yet to be conducted to understand the mechanisms involved in the biological response to implant wear debris. A comprehensive understanding of the innate immune system can support the creation of therapeutic modalities to control inflammation and consequent implant failure.

## 8. Potential Biological Treatments to Prevent Implant Failure

A multitude of pharmacological methods has been researched to reduce implant debris-induced osteolysis. Some strategies include reducing the activity of pro-inflammatory cytokines, blocking the NF-kB pathway, and modulating macrophage polarization.

As previously mentioned, macrophage response to implant particles leads to a release of a host of cytokines and chemokines that elicit osteolysis and implant failure. Inhibiting this reaction may mitigate the inflammation in the periprosthetic tissue. TNF-α is a critical pro-inflammatory mediator in wear-induced inflammation, and its inhibition has been researched as a potential therapeutic target.

Progranulin, a peptide that antagonizes the binding of TNF-α to TNFR1/2, inhibited the inflammatory response against titanium particles in an air pouch model as well as osteoclastogenesis and osteolysis in vivo, ex vivo, and in vitro [69,70]. Furthermore, it was revealed that these results were achieved by inhibiting the NFkB/TNF-α pathway [70]. The soluble TNF-α antagonist, Etanercept, was shown to mitigate osteolysis induced by titanium wear debris in vitro [71].

Blockage of titanium particle-induced osteolysis using adalimumab (anti-TNFα), anakinra (anti-IL-1β), and tocilizumab (anti-IL-6) antibodies in vivo effectively abolished titanium-induced osteolysis [72]. Resveratrol, a plant compound, has been reported to have antioxidant properties, and its protective effect against titanium-induced wear debris was accomplished by reducing gene expression of TNF-α. Resveratrol has been shown to decrease phosphorylation of NF-kB, nitric oxide production, reactive oxygen species generation, and lipid peroxidation [73]. Overall, these studies collectively support TNF-α antagonists as a potential therapeutic approach, however, further investigation is still much needed to establish the efficacy and translatability to a clinical setting.

The NF-kB pathway is activated in macrophages when exposed to implant debris, suggesting that the modulation of this mechanism can have a robust therapeutic impact in preventing implant failure [12,27]. Osteoprotegerin (OPG), a decoy protein that prevents osteoclast activation, was investigated using a recombinant adeno-associated virus vector expressing OPG in a titanium-implanted mouse model. The results revealed a single intramuscular injection effectively produced high levels of OPG in myocytes, thereby inhibiting wear debris-induced osteolysis [74]. Similarly, targeting the NF-kB pathway using a NF-kB Decoy Oligodeoxynucleotide (ODN) was shown to limit debris-induced osteolysis in a mouse model with continuous femoral particle infusion. The NF-kB decoy ODN inhibits transcription factors from binding to promotor regions of specific genes in the NF-kB pathway. This effectively reversed UHMWPE-induced bone loss and decreased osteoclast numbers in a murine model infused with UHMWPE particles in the distal femur [75]. Research showed that local injections with NF-kB decoy ODNs in a murine model mitigated the UHMWPE-induced expression of RANK-L and TNF-α while inducing the expression of anti-resorptive cytokines [76].

Regulating IKB Kinase (IKK) was identified as another potential mechanism to inhibit the NF-kB pathway. A short peptide NEMO-Binding Domain (NBD) inhibited NF-kB activation by reducing IKK complex assembly, ultimately inhibiting RANK-L-induced osteolysis [77]. Moreover, NBD peptide appeared to reduce PMMA-induced NF-kB activation in a mouse calvarial model by inhibiting PMMA-induced osteolysis [78]. Furthermore, treatments with RANK: FC fusion protein, a recombinant RANK-L antagonist, inhibited bone resorption in a titanium-implanted mouse model [79]. A recent study also revealed Tussilagone farfara, a natural compound, inhibited osteoclastogenesis in a titanium particle-induced calvarial model. Tussilagone farfara was shown to inhibit the p38 MAP Kinase and NF-kB signaling pathways, thereby serving as a potential agent to prevent periprosthetic osteolysis-induced aseptic loosening [80]. Collectively, these studies reveal NF-kB signaling as a potential target for future therapeutic strategies in preventing implant debris-induced osteolysis. Further studies, however, are needed to delineate the safety of these agents in a clinical setting.

Osteoclast activity can be regulated to avoid bone resorption using bisphosphonates. These drugs suppress osteoclast precursors from differentiating and can also promote macrophage apoptosis [81].

The inhibiting effect of Pamidronate, a member of the bisphosphonate family, on UHMWPE-induced TNF-α release from murine macrophages was studied. Pamidronate suppresses the PMMA-induced bone resorption in a co-culture model of murine calvaria and macrophages [82,83]. Similarly, bisphosphonate, disodium ethane-1, 1-diphosphonate (EHDP) abolished osteoclast differentiation and subsequent osteolysis in a murine monocytes and macrophages co-culture model derived from granulomas formed by subcutaneous implantation of PMMA, titanium, and UHMWPE particles with osteoclasts embedded on bone slices [84]. A meta-analysis of clinical trials on administering bisphosphonates post-operatively revealed short and mid-term anti-osteolytic effects in periprosthetic bone in patients who have undergone arthroplasty [85–87]. This supported the potential benefits of bisphosphonates in prosthesis-induced bone resorption. Additionally, bisphosphonates were shown to mitigate periprosthetic osteolysis 5 to 10 years after total joint arthroplasty [87]. Bisphosphonates may be a potential strategy for reducing aseptic loosening due to PPOL by preventing bone resorption. However, severe side effects such as femoral fractures and osteonecrosis of the jaw mitigate bisphosphonates use in a clinical setting in the context of aseptic loosening [88].

## 9. Conclusion

In recent decades, the immunological reaction to implant wear particles has been researched, and macrophages have been identified as the offending cells. The precise biological mechanism by which wear debris induces macrophages has yet to be fully understood. Recent research reveals wear particles from implants are identified by several PRRs. Activation of TLRs has also been elucidated as one of the most integral mechanisms in macrophage reactivity to prosthetic debris. The initiation of these mechanisms generates an inflammatory response that promotes aseptic loosening and ultimately implant failure.

Currently, no biological treatments effectively manage the immune response to periprosthetic debris. However, several therapies have been identified in ongoing research, including neutralizing pro-inflammatory cytokines, inhibiting the NF-kB pathway, and using bisphosphonates to modulate osteoclast activity. Further research has yet to be conducted to assess their safety and therapeutic efficacy. A thorough understanding of the biological mechanisms involved in implant wear debris, and the innate immune system will further support identifying potential targets for future therapeutic models.

## Figures and Tables

**Table 1:** Orthopedic prosthetic debris act on different immune cells around the implant inducing the release of various cytokines. Different chemokines recruit different immune cells. This schematic highlights the various chemokines that are best targeted for reducing implant-induced inflammation.

Implant debris-activated immune cells to release chemokines	Debris-induced inflammatory chemokines	Receptor	Site of the receptor
Macrophage (with TLR receptors)	IL-8 (CXCL8)	CXCR1	Macrophage, Neutrophils
Macrophage	MCP-1 (CCL2)	CCR2	Monocytes, NK cells, T cells, B cells, Fibroblasts
	MCP-4 (CCL13)		
	MCP-3 (CCL7)		
	MCP-2 (CCL8)		
Lymphocytes	TARC (CCL17)	CCR4	Monocytes, NK cells, Th2 cells
Neutrophils, Lymphocytes	MIP-3α (CCL20)	CCR6	Dendritic Cells, T cells, B cells
Monocytes	MIP-1β (CCL4)	CCR5	Monocytes, Macrophages, NK cells

MCP, monocyte chemoattractant protein; MIP, macrophage inflammatory protein; TARC, thymus and activation related chemokine.

## Data Availability

Not applicable since the information is gathered from published articles.
